# Antenatal depressive symptoms in rwanda: rates, risk factors, and social support

**DOI:** 10.1186/s12884-022-04522-4

**Published:** 2022-03-08

**Authors:** Marie Providence Umuziga, Darius Gishoma, Michaela Hynie, Laetitia Nyirazinyoye

**Affiliations:** 1 School of Nursing, College of Medicine and Health Sciences, University of Rwanda, P.O. Box 3286, Kigali, Rwanda; 2 Department of Psychology, York University, 4700 Keele Street, Toronto, ON M3J 1P3 Canada

**Keywords:** Antenatal depression, Maternity Social Support Scale (MSSS), Pregnancy, Rwanda

## Abstract

**Background:**

Prevalence of perinatal depression is high in Rwanda and has been found to be associated with the quality of relationship with partner. This study extends this work to examine the relationship between antenatal depressive symptoms and social support across several relationships among women attending antenatal care services.

**Methods:**

Structured survey interviews were conducted with 396 women attending antenatal care services in 4 health centres in the Southern Province of Rwanda. The Edinburgh Postnatal Depression Scale (EPDS) and Maternity Social Support Scale (MSSS) were used to assess antenatal depressive symptoms and the level of support respectively. Socio-demographic and gestational information, pregnancy intentions, perceived general health status, and experience of violence were also collected. Univariate, bivariate analyses and a multivariate logistic regression model were performed to determine the relationship between social support and risk factors for antenatal depressive symptoms.

**Results:**

More than half of respondents were married (55.1%) or living with a partner in a common-law relationship (28.5%). About a third (35.9%) were in their 6th month of pregnancy; the rest were in their third term. The prevalence of antenatal depressive symptoms was 26.6% (EPDS ≥ 12). Bivariate analyses suggested that partner and friend support negatively predict depression level symptoms. Adjusting for confounding variables such as unwanted pregnancy (AOR: 0.415, CI: 0.221- 0.778), parity (AOR: 0.336, CI: 0.113–1.000) and exposure to extremely stressful life events (AOR: 2.300, CI: 1.263- 4.189), partner support (AOR: 4.458, CI: 1.833- 10.842) was strongly significantly associated with antenatal depressive symptoms; women reporting good support were less likely to report depressive symptoms than those reporting poor support or those with no partner. Friend support was no longer significant.

**Conclusion:**

The study revealed that social support may be a strong protector against antenatal depressive symptoms but only support from the partner. This suggests that strengthening support to pregnant women may be a successful strategy for reducing the incidence or severity of maternal mental health problems, but more work is required to assess whether support from the broader social network can compensate for absent or unsupportive partners.

## Background

Despite the overriding expectation of pregnancy as a joyous occasion, it is a time of dynamic changes, full of anticipation and preparation. Unfortunately, for many women, pregnancy is also a catalyst for the onset of a new depressive disorder or a precipitant for recurrent depression [1, 2]. Perinatal mental health issues constitute an important public health problem, having a significant impact on the mother, the family, the husband or partner, mother-baby interactions, and on the long-term emotional, and cognitive development of the baby [2, 3]. Antenatal depression is a major health burden and is the most prevalent mental disorder in the perinatal period [4]. It is a non-psychotic depressive episode that occurs in the last trimester of pregnancy and is characterized by mild to severe symptoms that occur during pregnancy. It is associated with decreased interest in almost all pleasurable activities, sad mood, hopelessness, sleep disturbance, fatigue, changes in appetite, suicidal ideation, feelings of worthlessness, lack of concentration, reduced self-esteem and confidence [3, 4]. Antenatal depression has also been found to be a risk factor for pre-term birth and low birth weight, infant under-nutrition, and stunting, as well as higher rates of diarrhoeal diseases [5]. Additionally, antenatal depressive symptoms have the potential to impact negatively upon health service utilization and thereby contribute to increased perinatal complications and maternal mortality [6]. There is a growing evidence that antenatal depression also leads to postnatal depression, and has influence on offspring’s cognitive development, emotions and behaviors in childhood [4, 7]. However, the magnitude and risk factors of maternal depression during pregnancy in developing countries are currently poorly understood [8], and antenatal depression is often overlooked in routine screening [9].

The prevalence estimates of antenatal depression can vary across regions globally [9]. However, the burden of antenatal depression is generally higher in low- and middle-income countries (LMICs). In LMICs, the prevalence of antenatal depression measured by screening or diagnostic tools is as high as 20–26% (11,12). A systematic review of studies conducted in LMICs reported the prevalence of common perinatal mental disorders (CPMDs) including antenatal depression to be 15.6% [6], but a study by Gelaye and colleagues reported that the pooled prevalence estimates of antenatal depression were as high as 25.3% in LMICs [5]. Thus, there is considerable disagreement about the prevalence of antenatal depression in LMICs, which may be the result of using different cut-off scores on depression scales, different populations (hospital versus community or clinic samples) and country-wide differences in risk factors. A systematic review of studies with women in Africa conducted in 2010 by Sawyer and colleagues reported estimate rates of antenatal depression to range between 12.5 to 27.1% [10]. Meanwhile, in a recent systematic and meta-analysis on the prevalence of antenatal depression in Africa the pooled prevalence of antenatal depression was reported to be 26.3% [8]. Thus, the variability in estimates is also observed across the African continent.

Mental health problems are prevalent in Rwanda, a small country of 12 million people located in East Africa. A recent 2018 Rwanda mental health survey assessed mental health using the Mini-International Neuropsychiatric Interview with a nationwide cross-sectional survey of 19,110 randomly selected individuals aged 14 to 65 years. The survey revealed a high prevalence of mental disorders in the general population, with depression among the most prevalent at 11.9% in the general population [11]. Given that a history of mental health problems is a risk factor for perinatal depression, these high rates of mental health issues suggest that high rates of antenatal depression may also be found. To date, however, there has been limited research on prevalence rates and risk factors for antenatal depression in Rwanda. A recent study by Umuziga and her colleagues found relatively high rates of women at risk of antenatal depressive symptoms (37.6%) in a sample of 165 perinatal women [12], but used cut off scores of over 10 on the Edinburgh Postnatal Depression Survey (EPDS) [13]. While these scores are associated with “possible depression”, most epidemiological studies of perinatal depression use higher cut-off scores associated with “probable depression” [13].

The elevated rates of antenatal depression in LMICs may be attributed to difficult living conditions experienced in these settings. This includes both material and social challenges and exposure to stressful life events. Known risk factors for maternal mental health problems repeatedly reported include poverty, low education levels, poor social support, and marital problems [7, 14, 15]. Dadi and colleagues added that antenatal depression is commonly associated with economic difficulties, bad obstetric history, poor support from relatives, previous mental health problems, and unfavourable marital conditions [14]. Consistent with other research on perinatal depression, both globally and in Africa more specifically, the study by Umuziga and colleagues in Rwanda found risk factors for antenatal depression included a poor relationship with one’s partner, having many children, being a very young mother (under 24), and exposure to stressful life events [12]. However, this study did not explicitly measure social support and did not look at other relationships beyond that with the partner. Moreover, many of the women in this study were visiting a hospital based neonatal clinic and so may not have been as representative of a community sample as a sample drawn from community health centres. Finally, the pregnant subsample in this study was relatively small, at only 85 women, further limiting generalizability.

Social support is a complex construct that can refer to the receipt of emotional, material or information resources (received support) or the perception that resources are available if needed (perceived support) [16]. Not only is social support found to be associated with perinatal depression but a recent study with mothers in Australia found that antenatal perceptions of social support are amenable to intervention, and that increased perceptions of support can reduce rates of postnatal depression among women at risk [17]. In Rwanda, a recent study has shown that perinatal depression is more likely in the face of a poor quality spousal relationship or difficulties with in-laws, or being a single mother [18]. Dennis also highlighted the importance of the quality of social relationships with people outside of the immediate family [19]. The goal of the present research was thus to obtain an estimate of rates of antenatal depression in women attending antenatal care (ANC) services in community clinics in different centres in the Southern Province and to explore the role of social support as a protective factor to the development and severity of antenatal depression.

## Methods

### Design, population and setting

A descriptive cross-sectional, health facility-based survey was conducted from June to July 2019. The study utilizes baseline data from a quasi-experimental study on the impact of health provider mentorship and peer support. G*Power software as a free power analysis program for variety of statistical tests commonly used in the social, behavioral, and biomedical sciences [20] was used to determine the sample size. With an effect size of 0.05, α error probability = 0.05, Power (1-β error probability) = 0.95, and 4 groups, the approximated sample size calculated was 74 per group. However, in anticipation of participant attrition we added 26 additional participants to each group. A total of 396 pregnant women receiving regular antenatal care who were in their 6^th^ month of pregnancy or above were recruited from four randomly selected health centres of Southern Province in Rwanda. Participants ranged in age from 16 to 45 years of age (M = 30.8 SD = 6.58).

The study sites were selected using simple random sampling by picking names from a box [21]. In total, four sites with matched characteristics were included in this study. All pregnant mothers attending their ANC services who were in their 6th month of pregnancy or later were invited to participate in the study, with the help of a midwife/nurse in charge of ANC. The researchers also used the ANC registry to identify women who were eligible but who did not have their appointment within the period of data collection in order to also include them. The Community health workers in charge of maternal and new born care affiliated with each health centre helped to reach those other women.

### Measures and data collection process

Data collection started after obtaining ethical approval from the Institutional Review Board of the College of Medicine and Health Sciences/ University of Rwanda and permission from the administrative districts of the study sites. All study methods were performed in accordance with Declaration of Helsinki ethical principles [22]. Written informed consent was obtained from all the participants and from their legal guardians or parents if participants are below 18 years of age (Age of majority in Rwanda) [23]. A structured questionnaire administered by one of researchers and research assistants was used to obtain socio-demographic information of participants, gestational and obstetric information, risk and protective factors, physical and mental health and maternal social support.

**Socio-demographic variables** included age, marital status, social-economic class, education and occupation. Socio-economic class (*ubudehe*), which is a social stratification programme determined by household income, has four categories [24]. None of the women were in the fourth, or highest, category so only the lowest three are reported.

**Gestational information and self-rated health** questions included, gestational age (number of months pregnant); type of last delivery (normal vaginal, vaginal with complications, caesarian section), sex preference for the infant; planned pregnancy (yes or no) and parity. Participants rated their perceived overall health on a one-item measure with a 5-point response option, ranging from very poor to very good.

#### Risk and protective factors

Participants were asked to indicate any prior exposure to violence or negative life events (e.g., experience of gender-based violence, childhood abuse, stranger violence, loss of any family member, etc.). This variable was recoded into a binary variable: having never experienced any of these events versus having experienced at least one stressful life event.^1^

#### Depressive symptoms

Antenatal depressive symptoms were assessed using the Edinburgh Post-Natal Depression Scale (EPDS) [13]. This tool has been widely used in sub-Saharan Africa and is valid and reliable for testing both severity and probability of depression during perinatal period in multiple countries and languages [25]. The EPDS has been validated as a screening tool for antenatal depression in the previous studies in Rwanda [18, 26] and was reported to be reliable tool for screening antenatal depressive symptoms in two other studies with Cronbach’s Alpha values over 0.80 [12, 18]. We followed the recommendation of Gureje and colleagues [9] to use a score of 12 or higher to define case level depressive symptoms (hereafter referred to as ‘depression’). The EPDS was also found to be reliable in this study, with a Cronbach’s Alpha value of 0.894.

**Social Support** was measured using a modified version of the Maternity Social Support Scale (MSSS), developed by Webster and colleagues [27]. The original scale is a brief 6-item survey measuring support from spouse, friends and family on a five-point Likert scale for each item. The current scale was expanded from 6 to 12 items to include additional supporters deemed relevant in the Rwandan context (in-laws, community members, neighbours). The Cronbach’s Alpha value was 0,739 for the full scale. An exploratory principal axis factor analysis, with oblimin rotation, was performed to confirm that the translated and revised scale measured spousal relationships, peer relationships and family relationships as intended. The best solution based on an analysis of the scree plot and eigen values generated four factors. The first factor, with an eigen value of 3.32, explained 30.17% of the variance. This factor described the relationship with husband/partner. Items loading on this factor included: my husband helps me a lot, there is conflict with husband, I feel loved by husband with factor loadings of 0.80, -0.77, 0.95 respectively. The second factor, with an eigen value of 1.77, explained 16.09% of the variance, and reflected support from friends/peers. The following items loaded on the second factor: I have good friends who support me, I have neighbours who support me, I have people in the community who support me, with factor loadings of 0.74, 0.86, 0.78, respectively. Conflict with nuclear family and extended family loaded onto a third factor with an eigen value of 1.33, which explained only 12.07% of the variance (loadings of 0.60, 0.60 respectively), and conflict with in-laws loaded onto a fourth factor (at 0.49), with an eigen value of 1.03, explaining 9.39% of the variance. Two items did not load onto any factor: support from nuclear family and control by husband. These items were therefore excluded. Moreover, the scores on the last two factors were highly skewed and so were excluded from further analyses. Maternal social support was therefore measured using the means of items from factor 1, husband/partner support, and factor 2, friend support, respectively. Scores were categorized into poor versus good support, based on the mid-point of the 5-point scale, with scores of 1 to 2 coded as poor support, and 3 to 5 coded as good support. For partner support, a third category of no partner was also added.

### Data analysis

The data were analyzed using SPSS version 21. Univariate, bivariate, and multivariate logistic regression analyses were performed to summarize the dependent and independent variables, and examine the relationship between the independent variables and antenatal depressive symptoms. The binary logistic regression analyses were conducted between statistically significant variables in the bivariate tests and antenatal depressive symptoms.

A multivariate logistic regression analysis was employed to identify independent risk factors of antenatal depression. Antenatal depression was coded as “1” for antenatal depressive symptoms (EPDS ≥ 12 scores) and “0” for the absence of depressive symptoms (EPDS < 12 scores). Variables were entered for multivariate logistic regression analysis if they achieved statistical significance (*p* < 0.05) in the bivariate analysis. Estimated associations were described using adjusted odds ratios (AOR) with 95% confidence intervals (CIs).

## Results

### Participants’ sample characteristics

Sample characteristics were determined using frequencies, means, and standard deviations and are reported in Table 1. The sample was comprised of 396 participants from age 16 to 45 years. About a half were married (55.1%) or living with a common-law partner (28.5%), the main occupation was farming and cultivating clops (77.3%) and only 32.8% completed primary school.

**Table 1:** Sample characteristics and factors associated with antenatal depressive symptoms among women attending antenatal care (ANC) services in Southern Province in Rwanda

Sample and socio-demographic characteristics		Total	Depressive symptoms (EPDS ≥ 12)	*P*-value
		**N (%)**	**N (%)**	
		**396 (100%)**	**105 (26.5%)**	
**Age (Mean: 30.8; SD: 6.58)**				
24 or less	95 (24)		28 (29.5)	0.354
25–34	186 (47)		43 (23.1)	
35 or more	115 (29)		34 (29.6)	
**Marital status**				
Single/separated/divorced/widow	65 (16.4)		32 (49.2)	< 0.001
Married	218 (55.1)		49 (22.5)	
Living together as spouses	113 (28.5)		24 (21.2)	
**Level of education**				
No education	20 (5.1)		4 (20)	0.166
Primary not completed	145 (36.6)		49(33.8)	
Primary completed	130 (32.8)		31 (23.8)	
High school not completed	52 (13.1)		14 (26.9)	
High school completed	29 (7.3)		5 (17.2)	
Vocational education	6 (1.5)		1(16.7)	
Tertiary	14 (3.5)		1 (7.1)	
**Women's occupation**				
Farming/cultivating crops	306 (77.3)		79 (25.8)	0.465
Employed	39 (9.8)		9 (23.1)	
Not employed	51 (12.9)		17 (33.3)	
**Socio-economic class (Ubudehe)**				
First category (lowest)	47 (11.9)		22 (46.8)	0.010
Second category	165 (41.7)		39 (23.6)	
Third category	181 (45.7)		43 (23.8)	
Missing	3 (0.8)		1 (33.3)	

As indicated in Table 2; a third of the pregnant mothers (35.9%) were in their late second term (6 months of pregnancy).

**Table 2:** Distribution of participants by women's gestational, health and social support information

Women's gestational and health information		Total N (%)	Depressive Symptoms N (%)	*P*-value
**Gestational age**				
6 months		142 (35.9)	37 (26.1)	0.797
7 months		102 (25.8)	24 (23.5)	
8 months		106 (26.8)	30 (28.3)	
9 months		46 (11.6)	14 (30.4)	
**Pregnancy intention**				
Wanted		296 (74.7)	52 (17.6)	< 0.001
Unwanted		100 (25.3)	53 (53)	
**Parity (number of children given birth to)**				
0		114 (28.8)	24 (21.1)	0.040
1 to 3		232 (58.6)	61 (26.3)	
4 and more		50 (12.6)	20 (40)	
**Type of delivery on last pregnancy**				
Normal vaginal delivery		199 (50.3)	57 (28.6)	0.325
Vaginal delivery with complications		31 (7.8)	11 (35.5)	
Caesarean section		53 (13.4)	13 (24.5)	
N/A (first pregnancy)		113 (28.5)	24 (24.2)	
**Mothers' perceived health status**				
Poor		73 (18.4)	48 (65.8)	< 0.001
Neither poor nor good		174 (43.9)	48 (27.6)	
Good		149 (37.6)	9 (6)	
**Have you lost a child**				
Yes	58 (14.6)		19 (32.8)	0.244
No	338 (85.4)		86 (25.4)	
**Size of the family (number of children taken care of)**				
No child	115 (29.1)		25 (21.7)	0.16
1–3	231 (58.3)		62 (26.8)	
4 and plus	50 (12.6)		18 (36)	
**Have you experienced a stressful event (checked off at least 1)**				
Yes	123 (31.1)		60 (48.8)	< 0.001
No	273 (68.9)		45 (16.5)	
**Maternal social support**				
**1. Husband/partner support**				
No partner	65 (16.4)		32 (49.2)	< 0.001
Poor support	109 (27.5)		59 (54.1)	
Good support	289 (72.5)		46 (16)	
**2. Friend support**				
Poor support	216 (54.5)		79 (36.6)	< 0.001
Good support	180 (45.5)		26 (14.4)	

### Prevalence of antenatal depressive symptoms

Scores on the Edinburgh Postnatal Depression Scale (EPDS) for the entire sample ranged from 0 to 30 (M = 8.86, SD = 6.501). Women who reported scores of 12 or greater on the EPDS were coded as having symptoms of antenatal depression. Table 1 provides the descriptive statistics for the overall sample, and for the subsample of women with antenatal depressive symptoms. For the overall sample, a total of 26.6% of pregnant women were found to have antenatal depressive symptoms. *P*-values below 0.05 were used to guide decisions for inclusion in the subsequent regression. Among socio-demographic variables, marital status (*p* < 0.001) and socioeconomic class (ubudehe) (*p* = 0.01) were found to predict antenatal depression. The Table 2 indicates that having experienced a stressful life event (*p* < 0.001) were found to have the most reliable relationship to depression level symptoms. For women’s gestational information, antenatal depressive symptoms were reliably associated with parity (*p* = 0.04), unplanned pregnancy (*p* < 0.001) and perceived health status (*p* < 0.001).

### Factors predicting the likelihood of antenatal depressive symptoms in Rwanda

The full model fit well, X^2^(8) = 135.103, *p* < 0.000, Nagerkerke R^2^_=_0.440. Findings illustrated in Table 3 indicate that parity is strongly associated with antenatal depressive symptoms; women with no child (primigravida) were less likely to have antenatal depressive symptoms than those with 4 or more children (Adjusted Odds Ratio (AOR): 0.336, Confidence interval (C.I.) = 0.113–1.000). Women who reported a wanted pregnancy were less likely to have antenatal depressive symptoms than those with an unwanted pregnancy (AOR: 0.415, CI: 0.221- 0.778).^2^ Respondents who reported having had any extremely stressful life events were more likely to have antenatal depressive symptoms (AOR = 2.300, C.I. = 1.263–4.189) than those who had not (see Table 3). In contrast, socio-economic class (ubudehe) did not show a statistically significant association in the multivariate analysis. As predicted, partner support had the largest effect sizes, showing strong relationships with antenatal depressive symptoms; both women with no partner (AOR: 4.458, CI: 1.833- 10.842) and those with poor husband/partner support were more likely to have antenatal depressive symptoms (AOR: 3.366, CI: 1.593- 7.113). In the multivariate analysis, friend support no longer showed a statistically significant association with depressive symptoms.

**Table 3:** Logistic regression predicting the likelihood of antenatal depressive symptoms among women in Southern Province in Rwanda

Independent variables	B	S.E	*P*-values	Odds ratio	95% C.I	
					**Lower**	**Upper**
**Ubudehe (socio-economic class)**			0.24			
First	0.654	0.439	0.136	1.924	0.814	4.546
Second	-0.066	0.316	0.835	0.936	0.504	1.74
Third (r)				1		
**Parity**			0.127			
Primigravida	-1.091	0.557	0.050	0.336	0.113	1
One child to 3	-0.390	0.424	0.357	0.677	0.295	1.553
4 and more (r)				1		
**Perceived health status**			0			
Poor	2.414	0.471	0	11.176	4.441	28.126
Neither poor nor good	1.445	0.416	0.001	4.24	1.876	9.587
Good (r)				1		
**Pregnancy desired**						
Wanted	-0.88	0.321	0.006	0.415	0.221	0.778
Unwanted (r)				1		
**Stressful life event**						
At least one	0.833	0.306	0.006	2.300	1.263	4.189
None (r)				1		
**Maternity social support**						
**Husband/partner support**			0			
No partner	1.495	0.453	0.001	4.458	1.833	10.842
Poor	1.214	0.382	0.001	3.366	1.593	7.113
Good (r)				1		
**Friend support**						
Poor	0.199	0.325	0.540	1.221	0.646	2.307
Good (r)				1		
Constant	-2.253	0.541	0	0.105		

*r* Reference category, *df* degree of freedom, *B* Regression coefficients

## Discussion

The findings of this study suggest that rates of antenatal depressive symptoms are high in Rwanda with more than one in four pregnant women in this study having scores in the depressive range. This study reveals a high prevalence of antenatal depressive symptoms compared to other African countries such as Malawi (19%) [1] and Ethiopia (16%) [28]. However, these findings are similar to the findings of a recent 2020 systematic review on prevalence of antenatal depression in Africa (26.3%)[14] and a previous unpublished community-based study in Rwanda (25.3%)[18].

The bivariate analyses showed that pregnant women who live without a partner (single, divorced/separated and widowed) were more susceptible to antenatal depression, consistent with the study by Woldetensay and colleagues [29]. However, the mere presence of a partner is not enough. Lack of support from partner was found to be important in the development of antenatal depressive symptoms, a finding consistent with previous research in Rwanda [12, 18, 26] and in a study by Faleschini and colleagues in Massachusetts [30]. This finding may be related to the impact of having an unwanted pregnancy, which has been verified as a risk factor for antenatal depression in Rwanda [12] and in other African countries such as Ethiopia [31]. Unplanned pregnancy affects partner support [31], and thus could have contributed to poor partner support in this study which was found to predict depressive symptoms among pregnant women. However, even if unwanted pregnancy increases women’s risk of depression, increased social support plays a protective role from depression [5]. Thus, identifying the wantedness of women’s pregnancies and the extent of social support they receive is needed during antenatal care visits to provide appropriate counselling and improve women’s mental health during pregnancy [17].

This research raises important questions about factors associated with mental health in social contexts with limited social and material resources. The results here suggest that women in highly vulnerable environments may be particularly dependent on support available from their immediate social networks, which likely includes both material and social support, and particularly the presence of a good relationship with a partner. However, the association of socio-economic class (ubudehe) and friend support with antenatal depressive symptoms was no longer apparent once the effects of other factors were controlled. Although this runs counter to findings in previous African studies that found a broad range of sociodemographic factors were associated with antenatal depression [25, 28], a study by Dibaba and colleagues in Ethiopia also found that sociodemographic factors such as age, family size, economic class, occupation and education were not significant [32].

The impact of poverty might not be associated with antenatal depressive symptoms in the Rwandan context due to the direct support provided by the Government of Rwanda to pregnant woman who are identified as being in the two lower ubudehe classes (1st and 2nd categories) [33]. This kind of support, “Shisha Kibondo flour for mothers,” is used to make a highly nutritious porridge for pregnant or breastfeeding women and could have played a protective role by reducing some of the stress of poverty and the known impacts of malnutrition on depression [34]. However, the impact of this kind of support needs to be verified in further studies.

Furthermore, the findings of this study, showed that parity was associated with antenatal depressive symptoms, primigravida women (who were expecting for the 1^st^ time) were less likely to have antenatal depressive symptoms than those that have given birth to 4 or more children. To mitigate this risk, expanding family planning services to reduce unplanned pregnancies and giving due attention to the mental wellbeing of pregnant mothers with a history of unwanted/unplanned pregnancy and women who have given birth to 4 or more children would help alleviate the short- and long-term consequences of antenatal depressive symptoms.

Finally, this research also explores the broad range of possible support sources mothers can draw on in the Rwandan context. The relative lack of impact of friend support once other factors are taken into account is not consistent with what has been found in other research, particularly in high income contexts [35]. It will be important to use qualitative research to understand how peer support is provided and experienced in the Rwandan context, in order to identify alternative sources of support for those women with poor or missing spousal support.

## Conclusion

The high rates of antenatal depressive symptoms among pregnant women attending antenatal care (ANC) services indicate that it is imperative to include screening for depression and its attendant risk factors to improve detection and referral for interventions. Integration of mental health service with ANC follow-up service and screening pregnant women for possible depression has paramount importance for a healthy pregnancy and prevention of the possible adverse health outcomes on the mother and the fetus. The importance of spousal support to maternal mental health has been noted elsewhere and reinforces the importance of considering maternal mental health at the level of the family and community, and not only as a factor affecting women alone.

## Data Availability

The datasets used and/or analysed during the current study are available from the corresponding author on reasonable request.

## Abbreviation

EPDS: Edinburgh Postnatal depression scale

## Footnotes

1 Three additional variables; whether pregnancy was planned or not, whether women were currently ill, and quality of relationship with husband were included in the survey but excluded from analyses as they were deemed to be redundant with whether the pregnancy was wanted, self-rated health and husband support, and the latter variables were deemed to be more important conceptually and/or measured using more sensitive questions.

2 Both wanted pregnancy and husband support remained important variables despite the fact that there was a strong relationship between the pregnancy being wanted and husband support, X^2^(2) = 40.98, p = 0.000. Among women with no partner, 53.8% had an unwanted pregnancy. Among those with a poor relationship, 33.9% said it was unwanted; among those with a good relationship it was only 16.7%.
